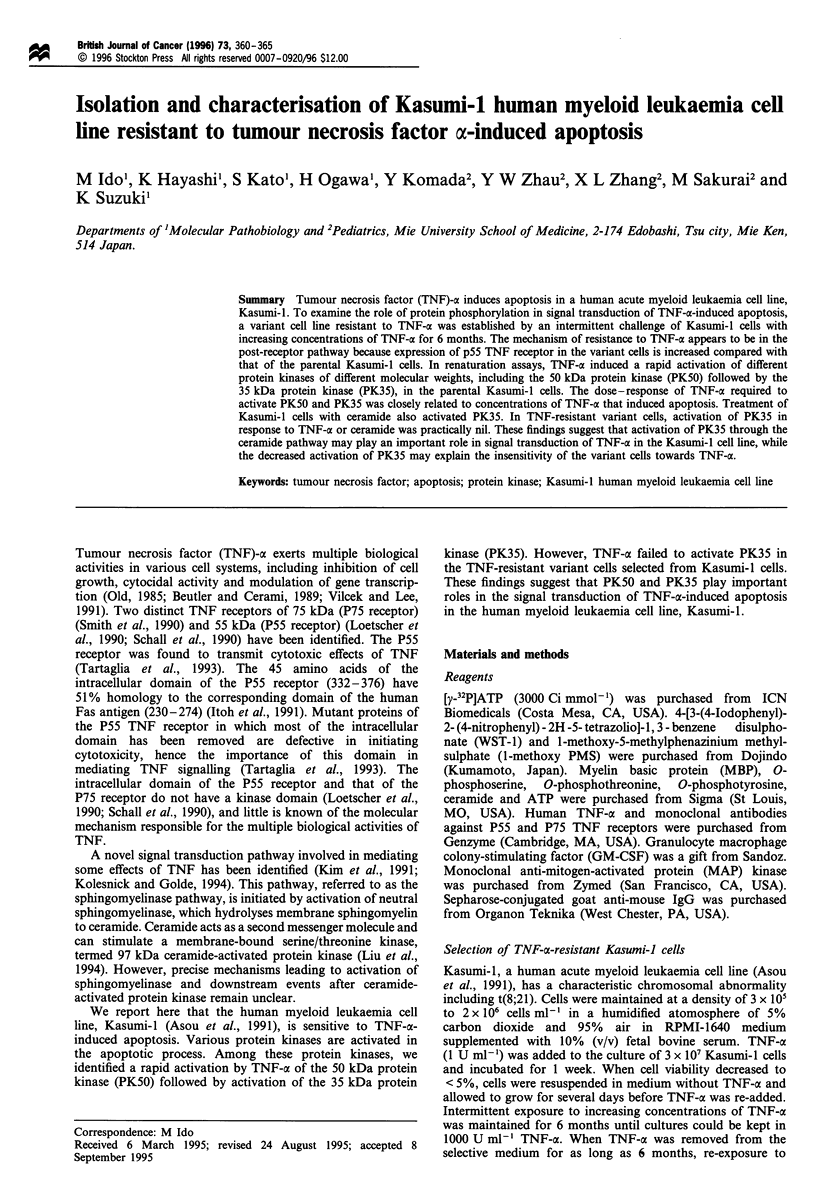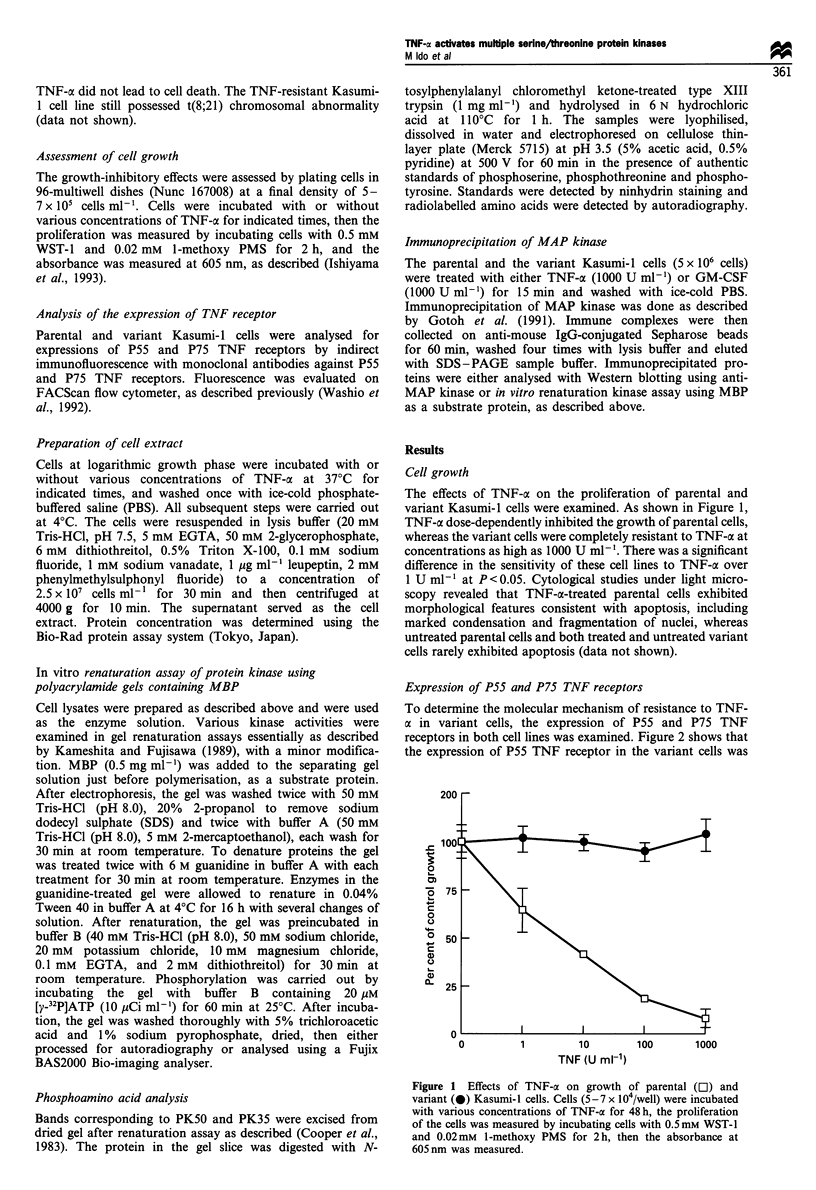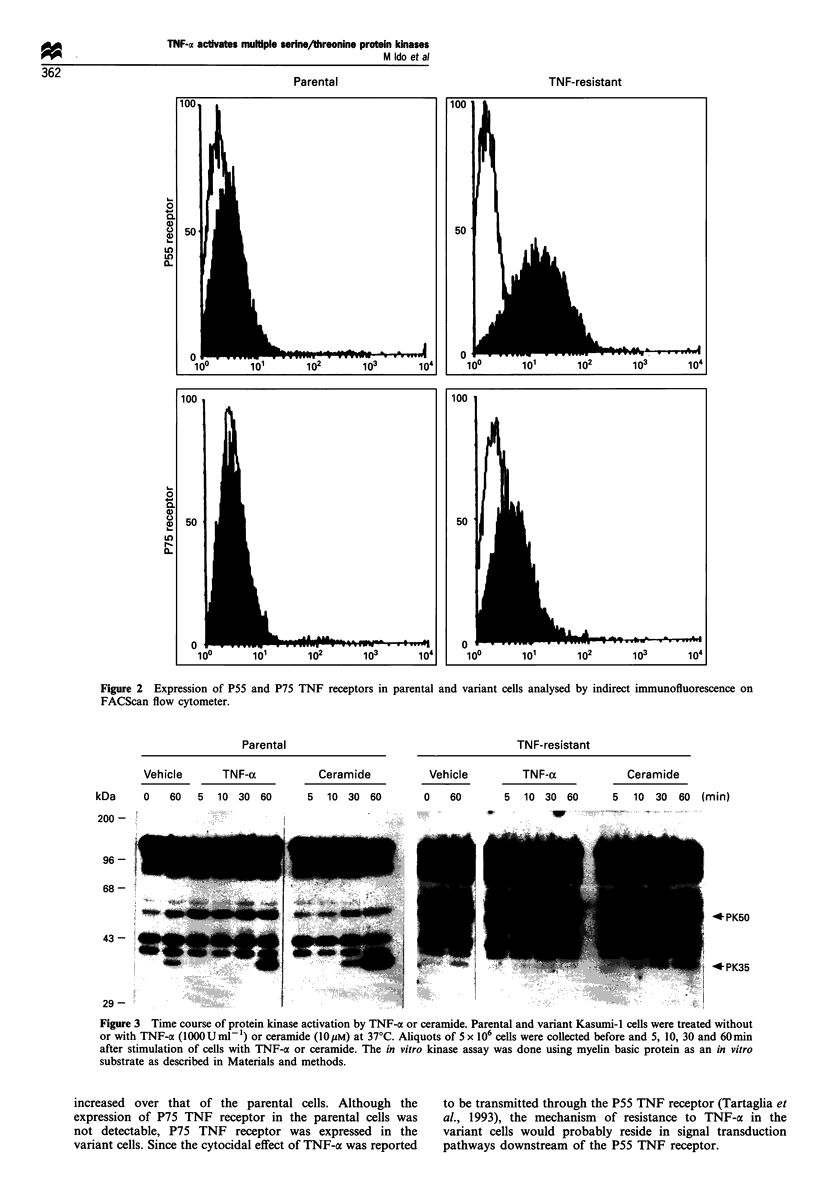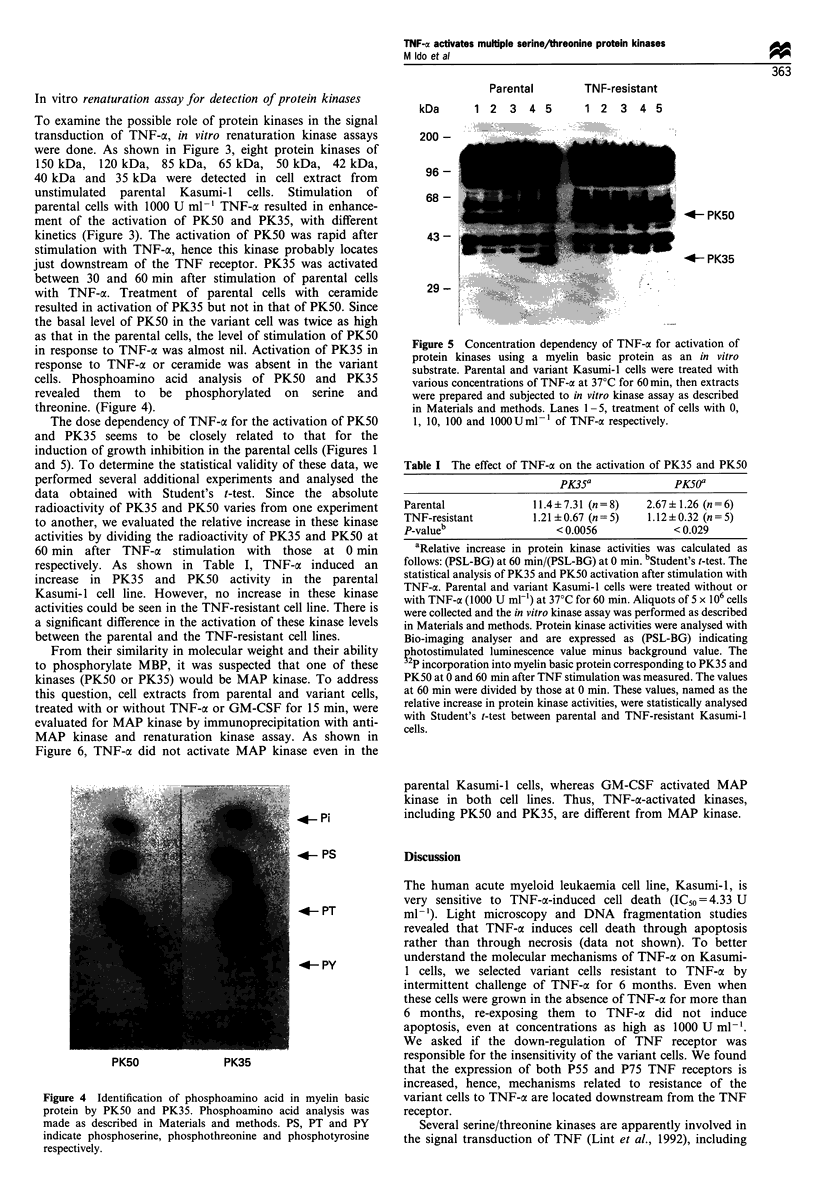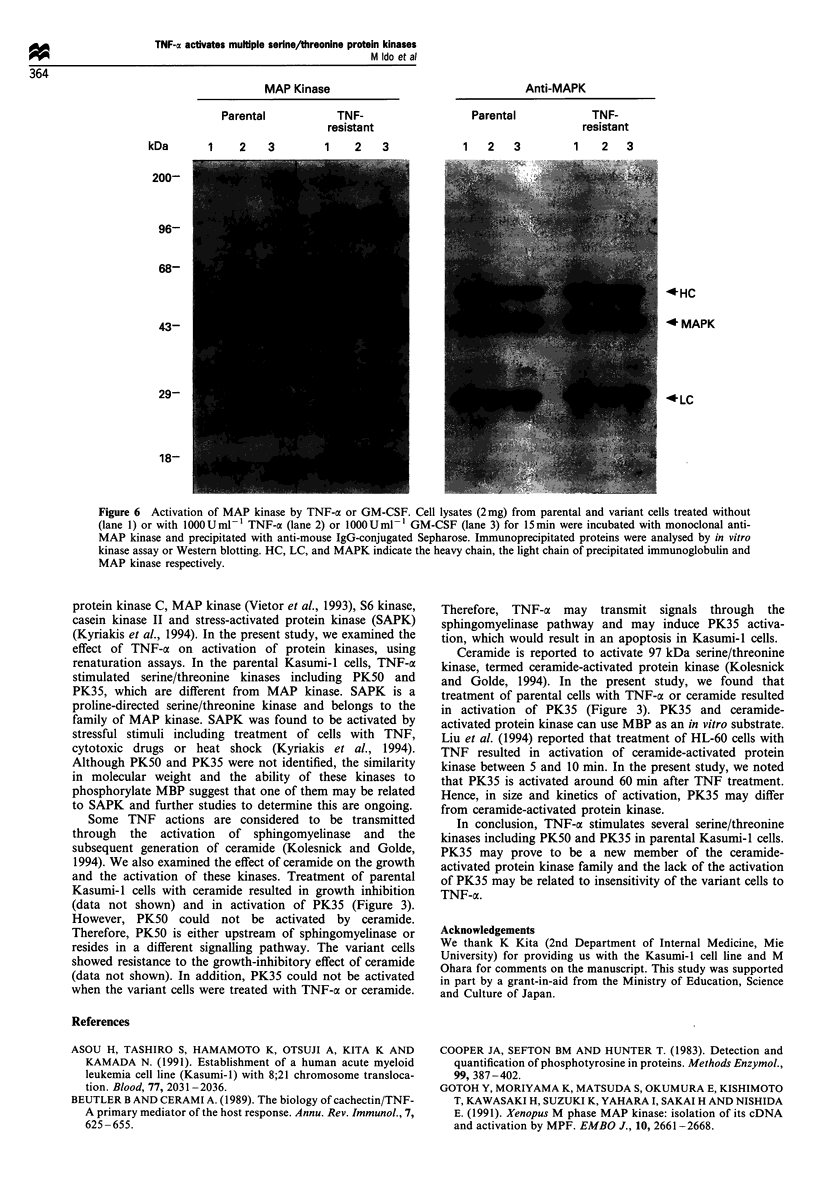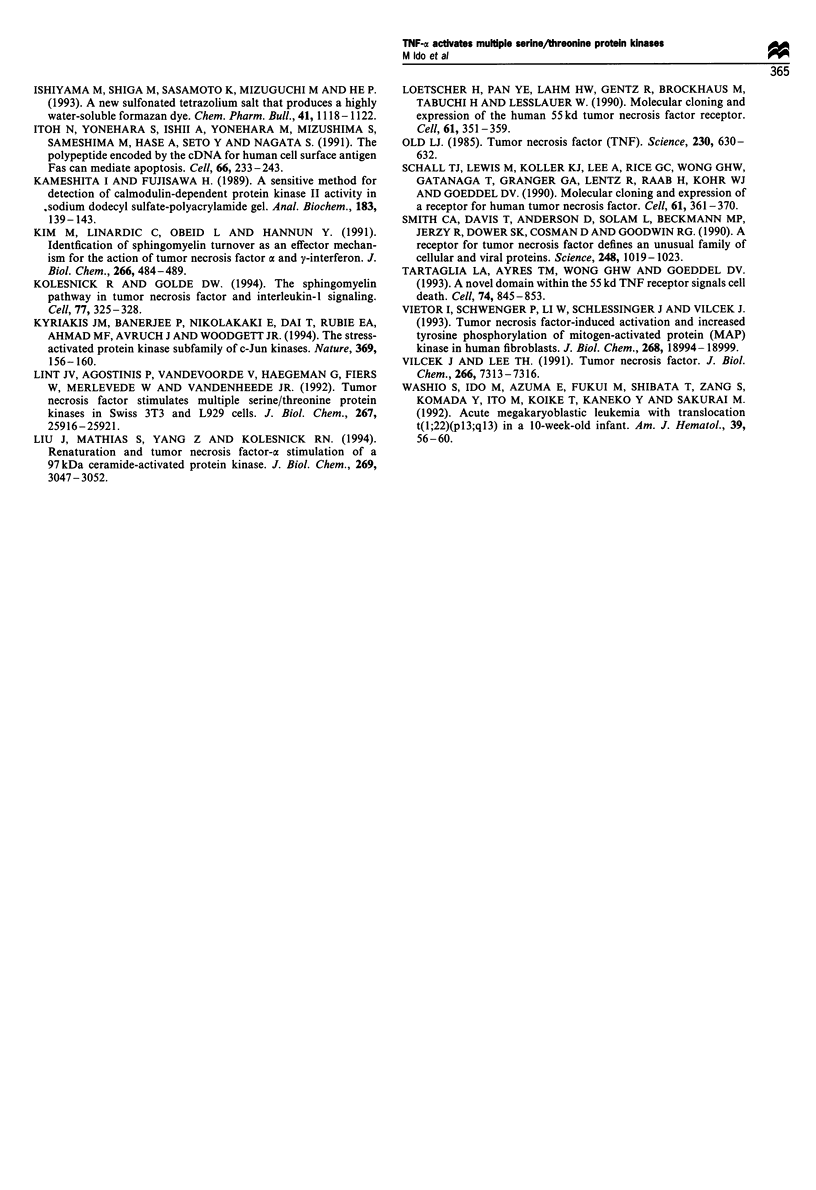# Isolation and characterisation of Kasumi-1 human myeloid leukaemia cell line resistant to tumour necrosis factor alpha-induced apoptosis.

**DOI:** 10.1038/bjc.1996.61

**Published:** 1996-02

**Authors:** M. Ido, K. Hayashi, S. Kato, H. Ogawa, Y. Komada, Y. W. Zhau, X. L. Zhang, M. Sakurai, K. Suzuki

**Affiliations:** Department of Molecular Pathobiology, Mie University School of Medicine, Japan.

## Abstract

**Images:**


British Journal of Cancer (1996) 73, 360-365
© 1996 Stockton Press All rights reserved 0007-0920/96 $12.00

### Isolation and characterisation of Kasumi-1 human myeloid leukaemia cell line resistant to tumour necrosis factor α-induced apoptosis

M Ido[1], K Hayashi[1], S Kato[1], H Ogawa[1], Y Komada[2], Y W Zhau[2], X L Zhang[2], M Sakurai[2] and K Suzuki[1]

*Departments of [1]Molecular Pathobiology and [2]Pediatrics, Mie University School of Medicine, 2-174 Edobashi, Tsu city, Mie Ken, 514 Japan.*

**Summary** Tumour necrosis factor (TNF)-α induces apoptosis in a human acute myeloid leukaemia cell line, Kasumi-1. To examine the role of protein phosphorylation in signal transduction of TNF-α-induced apoptosis, a variant cell line resistant to TNF-α was established by an intermittent challenge of Kasumi-1 cells with increasing concentrations of TNF-α for 6 months. The mechanism of resistance to TNF-α appears to be in the post-receptor pathway because expression of p55 TNF receptor in the variant cells is increased compared with that of the parental Kasumi-1 cells. In renaturation assays, TNF-α induced a rapid activation of different protein kinases of different molecular weights, including the 50 kDa protein kinase (PK50) followed by the 35 kDa protein kinase (PK35), in the parental Kasumi-1 cells. The dose-response of TNF-α required to activate PK50 and PK35 was closely related to concentrations of TNF-α that induced apoptosis. Treatment of Kasumi-1 cells with ceramide also activated PK35. In TNF-resistant variant cells, activation of PK35 in response to TNF-α or ceramide was practically nil. These findings suggest that activation of PK35 through the ceramide pathway may play an important role in signal transduction of TNF-α in the Kasumi-1 cell line, while the decreased activation of PK35 may explain the insensitivity of the variant cells towards TNF-α.

**Keywords:** tumour necrosis factor; apoptosis; protein kinase; Kasumi-1 human myeloid leukaemia cell line

Tumour necrosis factor (TNF)-α exerts multiple biological activities in various cell systems, including inhibition of cell growth, cytocidal activity and modulation of gene transcription (Old, 1985; Beutler and Cerami, 1989; Vilcek and Lee, 1991). Two distinct TNF receptors of 75 kDa (P75 receptor) (Smith *et al.*, 1990) and 55 kDa (P55 receptor) (Loetscher *et al.*, 1990; Schall *et al.*, 1990) have been identified. The P55 receptor was found to transmit cytotoxic effects of TNF (Tartaglia *et al.*, 1993). The 45 amino acids of the intracellular domain of the P55 receptor (332-376) have 51% homology to the corresponding domain of the human Fas antigen (230-274) (Itoh *et al.*, 1991). Mutant proteins of the P55 TNF receptor in which most of the intracellular domain has been removed are defective in initiating cytotoxicity, hence the importance of this domain in mediating TNF signalling (Tartaglia *et al.*, 1993). The intracellular domain of the P55 receptor and that of the P75 receptor do not have a kinase domain (Loetscher *et al.*, 1990; Schall *et al.*, 1990), and little is known of the molecular mechanism responsible for the multiple biological activities of TNF.

A novel signal transduction pathway involved in mediating some effects of TNF has been identified (Kim *et al.*, 1991; Kolesnick and Golde, 1994). This pathway, referred to as the sphingomyelinase pathway, is initiated by activation of neutral sphingomyelinase, which hydrolyses membrane sphingomyelin to ceramide. Ceramide acts as a second messenger molecule and can stimulate a membrane-bound serine/threonine kinase, termed 97 kDa ceramide-activated protein kinase (Liu *et al.*, 1994). However, precise mechanisms leading to activation of sphingomyelinase and downstream events after ceramide-activated protein kinase remain unclear.

We report here that the human myeloid leukaemia cell line, Kasumi-1 (Asou *et al.*, 1991), is sensitive to TNF-α-induced apoptosis. Various protein kinases are activated in the apoptotic process. Among these protein kinases, we identified a rapid activation by TNF-α of the 50 kDa protein kinase (PK50) followed by activation of the 35 kDa protein kinase (PK35). However, TNF-α failed to activate PK35 in the TNF-resistant variant cells selected from Kasumi-1 cells. These findings suggest that PK50 and PK35 play important roles in the signal transduction of TNF-α-induced apoptosis in the human myeloid leukaemia cell line, Kasumi-1.

### Materials and methods

### *Reagents*

[γ-$^{32}$P]ATP (3000 Ci mmol$^{-1}$) was purchased from ICN Biomedicals (Costa Mesa, CA, USA). 4-[3-(4-Iodophenyl)-2-(4-nitrophenyl)-2H-5-tetrazolio]-1,3-benzene disulphonate (WST-1) and 1-methoxy-5-methylphenazinium methylsulphate (1-methoxy PMS) were purchased from Dojindo (Kumamoto, Japan). Myelin basic protein (MBP), *O*-phosphoserine, *O*-phosphothreonine, *O*-phosphotyrosine, ceramide and ATP were purchased from Sigma (St Louis, MO, USA). Human TNF-α and monoclonal antibodies against P55 and P75 TNF receptors were purchased from Genzyme (Cambridge, MA, USA). Granulocyte macrophage colony-stimulating factor (GM-CSF) was a gift from Sandoz. Monoclonal anti-mitogen-activated protein (MAP) kinase was purchased from Zymed (San Francisco, CA, USA). Sepharose-conjugated goat anti-mouse IgG was purchased from Organon Teknika (West Chester, PA, USA).

### *Selection of TNF-α-resistant Kasumi-1 cells*

Kasumi-1, a human acute myeloid leukaemia cell line (Asou *et al.*, 1991), has a characteristic chromosomal abnormality including t(8;21). Cells were maintained at a density of $3 \times 10^5$ to $2 \times 10^6$ cells ml$^{-1}$ in a humidified atomosphere of 5% carbon dioxide and 95% air in RPMI-1640 medium supplemented with 10% (v/v) fetal bovine serum. TNF-α (1 U ml$^{-1}$) was added to the culture of $3 \times 10^7$ Kasumi-1 cells and incubated for 1 week. When cell viability decreased to < 5%, cells were resuspended in medium without TNF-α and allowed to grow for several days before TNF-α was re-added. Intermittent exposure to increasing concentrations of TNF-α was maintained for 6 months until cultures could be kept in 1000 U ml$^{-1}$ TNF-α. When TNF-α was removed from the selective medium for as long as 6 months, re-exposure to

Correspondence: M Ido
Received 6 March 1995; revised 24 August 1995; accepted 8 September 1995

TNF-α did not lead to cell death. The TNF-resistant Kasumi-1 cell line still possessed t(8;21) chromosomal abnormality (data not shown).

*Assessment of cell growth*

The growth-inhibitory effects were assessed by plating cells in 96-multiwell dishes (Nunc 167008) at a final density of 5–$7 \times 10^5$ cells $ml^{-1}$. Cells were incubated with or without various concentrations of TNF-α for indicated times, then the proliferation was measured by incubating cells with 0.5 mM WST-1 and 0.02 mM 1-methoxy PMS for 2 h, and the absorbance was measured at 605 nm, as described (Ishiyama *et al.*, 1993).

*Analysis of the expression of TNF receptor*

Parental and variant Kasumi-1 cells were analysed for expressions of P55 and P75 TNF receptors by indirect immunofluorescence with monoclonal antibodies against P55 and P75 TNF receptors. Fluorescence was evaluated on FACScan flow cytometer, as described previously (Washio *et al.*, 1992).

*Preparation of cell extract*

Cells at logarithmic growth phase were incubated with or without various concentrations of TNF-α at 37°C for indicated times, and washed once with ice-cold phosphate-buffered saline (PBS). All subsequent steps were carried out at 4°C. The cells were resuspended in lysis buffer (20 mM Tris-HCl, pH 7.5, 5 mM EGTA, 50 mM 2-glycerophosphate, 6 mM dithiothreitol, 0.5% Triton X-100, 0.1 mM sodium fluoride, 1 mM sodium vanadate, 1 $\mu g\ ml^{-1}$ leupeptin, 2 mM phenylmethylsulphonyl fluoride) to a concentration of $2.5 \times 10^7$ cells $ml^{-1}$ for 30 min and then centrifuged at 4000 g for 10 min. The supernatant served as the cell extract. Protein concentration was determined using the Bio-Rad protein assay system (Tokyo, Japan).

In vitro *renaturation assay of protein kinase using polyacrylamide gels containing MBP*

Cell lysates were prepared as described above and were used as the enzyme solution. Various kinase activities were examined in gel renaturation assays essentially as described by Kameshita and Fujisawa (1989), with a minor modification. MBP (0.5 mg $ml^{-1}$) was added to the separating gel solution just before polymerisation, as a substrate protein. After electrophoresis, the gel was washed twice with 50 mM Tris-HCl (pH 8.0), 20% 2-propanol to remove sodium dodecyl sulphate (SDS) and twice with buffer A (50 mM Tris-HCl (pH 8.0), 5 mM 2-mercaptoethanol), each wash for 30 min at room temperature. To denature proteins the gel was treated twice with 6 M guanidine in buffer A with each treatment for 30 min at room temperature. Enzymes in the guanidine-treated gel were allowed to renature in 0.04% Tween 40 in buffer A at 4°C for 16 h with several changes of solution. After renaturation, the gel was preincubated in buffer B (40 mM Tris-HCl (pH 8.0), 50 mM sodium chloride, 20 mM potassium chloride, 10 mM magnesium chloride, 0.1 mM EGTA, and 2 mM dithiothreitol) for 30 min at room temperature. Phosphorylation was carried out by incubating the gel with buffer B containing 20 $\mu$M [$\gamma$-$^{32}$P]ATP (10 $\mu Ci\ ml^{-1}$) for 60 min at 25°C. After incubation, the gel was washed thoroughly with 5% trichloroacetic acid and 1% sodium pyrophosphate, dried, then either processed for autoradiography or analysed using a Fujix BAS2000 Bio-imaging analyser.

*Phosphoamino acid analysis*

Bands corresponding to PK50 and PK35 were excised from dried gel after renaturation assay as described (Cooper *et al.*, 1983). The protein in the gel slice was digested with *N*-tosylphenylalanyl chloromethyl ketone-treated type XIII trypsin (1 mg $ml^{-1}$) and hydrolysed in 6 N hydrochloric acid at 110°C for 1 h. The samples were lyophilised, dissolved in water and electrophoresed on cellulose thin-layer plate (Merck 5715) at pH 3.5 (5% acetic acid, 0.5% pyridine) at 500 V for 60 min in the presence of authentic standards of phosphoserine, phosphothreonine and phosphotyrosine. Standards were detected by ninhydrin staining and radiolabelled amino acids were detected by autoradiography.

*Immunoprecipitation of MAP kinase*

The parental and the variant Kasumi-1 cells ($5 \times 10^6$ cells) were treated with either TNF-α (1000 U $ml^{-1}$) or GM-CSF (1000 U $ml^{-1}$) for 15 min and washed with ice-cold PBS. Immunoprecipitation of MAP kinase was done as described by Gotoh *et al.* (1991). Immune complexes were then collected on anti-mouse IgG-conjugated Sepharose beads for 60 min, washed four times with lysis buffer and eluted with SDS–PAGE sample buffer. Immunoprecipitated proteins were either analysed with Western blotting using anti-MAP kinase or *in vitro* renaturation kinase assay using MBP as a substrate protein, as described above.

### Results

*Cell growth*

The effects of TNF-α on the proliferation of parental and variant Kasumi-1 cells were examined. As shown in Figure 1, TNF-α dose-dependently inhibited the growth of parental cells, whereas the variant cells were completely resistant to TNF-α at concentrations as high as 1000 U $ml^{-1}$. There was a significant difference in the sensitivity of these cell lines to TNF-α over 1 U $ml^{-1}$ at $P < 0.05$. Cytological studies under light microscopy revealed that TNF-α-treated parental cells exhibited morphological features consistent with apoptosis, including marked condensation and fragmentation of nuclei, whereas untreated parental cells and both treated and untreated variant cells rarely exhibited apoptosis (data not shown).

*Expression of P55 and P75 TNF receptors*

To determine the molecular mechanism of resistance to TNF-α in variant cells, the expression of P55 and P75 TNF receptors in both cell lines was examined. Figure 2 shows that the expression of P55 TNF receptor in the variant cells was

**Figure 1** Effects of TNF-α on growth of parental (□) and variant (●) Kasumi-1 cells. Cells (5–$7 \times 10^4$/well) were incubated with various concentrations of TNF-α for 48 h, the proliferation of the cells was measured by incubating cells with 0.5 mM WST-1 and 0.02 mM 1-methoxy PMS for 2 h, then the absorbance at 605 nm was measured.

**Figure 2** Expression of P55 and P75 TNF receptors in parental and variant cells analysed by indirect immunofluorescence on FACScan flow cytometer.

**Figure 3** Time course of protein kinase activation by TNF-α or ceramide. Parental and variant Kasumi-1 cells were treated without or with TNF-α (1000 U $ml^{-1}$) or ceramide (10 μM) at 37°C. Aliquots of $5 \times 10^6$ cells were collected before and 5, 10, 30 and 60 min after stimulation of cells with TNF-α or ceramide. The *in vitro* kinase assay was done using myelin basic protein as an *in vitro* substrate as described in Materials and methods.

increased over that of the parental cells. Although the expression of P75 TNF receptor in the parental cells was not detectable, P75 TNF receptor was expressed in the variant cells. Since the cytocidal effect of TNF-α was reported to be transmitted through the P55 TNF receptor (Tartaglia *et al.*, 1993), the mechanism of resistance to TNF-α in the variant cells would probably reside in signal transduction pathways downstream of the P55 TNF receptor.

### In vitro *renaturation assay for detection of protein kinases*

To examine the possible role of protein kinases in the signal transduction of TNF-α, *in vitro* renaturation kinase assays were done. As shown in Figure 3, eight protein kinases of 150 kDa, 120 kDa, 85 kDa, 65 kDa, 50 kDa, 42 kDa, 40 kDa and 35 kDa were detected in cell extract from unstimulated parental Kasumi-1 cells. Stimulation of parental cells with 1000 U $ml^{-1}$ TNF-α resulted in enhancement of the activation of PK50 and PK35, with different kinetics (Figure 3). The activation of PK50 was rapid after stimulation with TNF-α, hence this kinase probably locates just downstream of the TNF receptor. PK35 was activated between 30 and 60 min after stimulation of parental cells with TNF-α. Treatment of parental cells with ceramide resulted in activation of PK35 but not in that of PK50. Since the basal level of PK50 in the variant cell was twice as high as that in the parental cells, the level of stimulation of PK50 in response to TNF-α was almost nil. Activation of PK35 in response to TNF-α or ceramide was absent in the variant cells. Phosphoamino acid analysis of PK50 and PK35 revealed them to be phosphorylated on serine and threonine. (Figure 4).

The dose dependency of TNF-α for the activation of PK50 and PK35 seems to be closely related to that for the induction of growth inhibition in the parental cells (Figures 1 and 5). To determine the statistical validity of these data, we performed several additional experiments and analysed the data obtained with Student's *t*-test. Since the absolute radioactivity of PK35 and PK50 varies from one experiment to another, we evaluated the relative increase in these kinase activities by dividing the radioactivity of PK35 and PK50 at 60 min after TNF-α stimulation with those at 0 min respectively. As shown in Table I, TNF-α induced an increase in PK35 and PK50 activity in the parental Kasumi-1 cell line. However, no increase in these kinase activities could be seen in the TNF-resistant cell line. There is a significant difference in the activation of these kinase levels between the parental and the TNF-resistant cell lines.

From their similarity in molecular weight and their ability to phosphorylate MBP, it was suspected that one of these kinases (PK50 or PK35) would be MAP kinase. To address this question, cell extracts from parental and variant cells, treated with or without TNF-α or GM-CSF for 15 min, were evaluated for MAP kinase by immunoprecipitation with anti-MAP kinase and renaturation kinase assay. As shown in Figure 6, TNF-α did not activate MAP kinase even in the parental Kasumi-1 cells, whereas GM-CSF activated MAP kinase in both cell lines. Thus, TNF-α-activated kinases, including PK50 and PK35, are different from MAP kinase.

**Figure 5** Concentration dependency of TNF-α for activation of protein kinases using a myelin basic protein as an *in vitro* substrate. Parental and variant Kasumi-1 cells were treated with various concentrations of TNF-α at 37°C for 60 min, then extracts were prepared and subjected to *in vitro* kinase assay as described in Materials and methods. Lanes 1–5, treatment of cells with 0, 1, 10, 100 and 1000 U $ml^{-1}$ of TNF-α respectively.

**Table I** The effect of TNF-α on the activation of PK35 and PK50

| | *PK35*[a] | *PK50*[a] |
|---|---|---|
| Parental | 11.4±7.31 (*n*=8) | 2.67±1.26 (*n*=6) |
| TNF-resistant | 1.21±0.67 (*n*=5) | 1.12±0.32 (*n*=5) |
| *P*-value[b] | <0.0056 | <0.029 |

[a]Relative increase in protein kinase activities was calculated as follows: (PSL-BG) at 60 min/(PSL-BG) at 0 min. [b]Student's *t*-test. The statistical analysis of PK35 and PK50 activation after stimulation with TNF-α. Parental and variant Kasumi-1 cells were treated without or with TNF-α (1000 U $ml^{-1}$) at 37°C for 60 min. Aliquots of $5 \times 10^6$ cells were collected and the *in vitro* kinase assay was performed as described in Materials and methods. Protein kinase activities were analysed with Bio-imaging analyser and are expressed as (PSL-BG) indicating photostimulated luminescence value minus background value. The $^{32}P$ incorporation into myelin basic protein corresponding to PK35 and PK50 at 0 and 60 min after TNF stimulation was measured. The values at 60 min were divided by those at 0 min. These values, named as the relative increase in protein kinase activities, were statistically analysed with Student's *t*-test between parental and TNF-resistant Kasumi-1 cells.

**Figure 4** Identification of phosphoamino acid in myelin basic protein by PK50 and PK35. Phosphoamino acid analysis was made as described in Materials and methods. PS, PT and PY indicate phosphoserine, phosphothreonine and phosphotyrosine respectively.

### Discussion

The human acute myeloid leukaemia cell line, Kasumi-1, is very sensitive to TNF-α-induced cell death ($IC_{50}$=4.33 U $ml^{-1}$). Light microscopy and DNA fragmentation studies revealed that TNF-α induces cell death through apoptosis rather than through necrosis (data not shown). To better understand the molecular mechanisms of TNF-α on Kasumi-1 cells, we selected variant cells resistant to TNF-α by intermittent challenge of TNF-α for 6 months. Even when these cells were grown in the absence of TNF-α for more than 6 months, re-exposing them to TNF-α did not induce apoptosis, even at concentrations as high as 1000 U $ml^{-1}$. We asked if the down-regulation of TNF receptor was responsible for the insensitivity of the variant cells. We found that the expression of both P55 and P75 TNF receptors is increased, hence, mechanisms related to resistance of the variant cells to TNF-α are located downstream from the TNF receptor.

Several serine/threonine kinases are apparently involved in the signal transduction of TNF (Lint *et al.*, 1992), including

**Figure 6** Activation of MAP kinase by TNF-α or GM-CSF. Cell lysates (2 mg) from parental and variant cells treated without (lane 1) or with 1000 U $ml^{-1}$ TNF-α (lane 2) or 1000 U $ml^{-1}$ GM-CSF (lane 3) for 15 min were incubated with monoclonal anti-MAP kinase and precipitated with anti-mouse IgG-conjugated Sepharose. Immunoprecipitated proteins were analysed by *in vitro* kinase assay or Western blotting. HC, LC, and MAPK indicate the heavy chain, the light chain of precipitated immunoglobulin and MAP kinase respectively.

protein kinase C, MAP kinase (Vietor *et al*., 1993), S6 kinase, casein kinase II and stress-activated protein kinase (SAPK) (Kyriakis *et al*., 1994). In the present study, we examined the effect of TNF-α on activation of protein kinases, using renaturation assays. In the parental Kasumi-1 cells, TNF-α stimulated serine/threonine kinases including PK50 and PK35, which are different from MAP kinase. SAPK is a proline-directed serine/threonine kinase and belongs to the family of MAP kinase. SAPK was found to be activated by stressful stimuli including treatment of cells with TNF, cytotoxic drugs or heat shock (Kyriakis *et al*., 1994). Although PK50 and PK35 were not identified, the similarity in molecular weight and the ability of these kinases to phosphorylate MBP suggest that one of them may be related to SAPK and further studies to determine this are ongoing.

Some TNF actions are considered to be transmitted through the activation of sphingomyelinase and the subsequent generation of ceramide (Kolesnick and Golde, 1994). We also examined the effect of ceramide on the growth and the activation of these kinases. Treatment of parental Kasumi-1 cells with ceramide resulted in growth inhibition (data not shown) and in activation of PK35 (Figure 3). However, PK50 could not be activated by ceramide. Therefore, PK50 is either upstream of sphingomyelinase or resides in a different signalling pathway. The variant cells showed resistance to the growth-inhibitory effect of ceramide (data not shown). In addition, PK35 could not be activated when the variant cells were treated with TNF-α or ceramide. Therefore, TNF-α may transmit signals through the sphingomyelinase pathway and may induce PK35 activation, which would result in an apoptosis in Kasumi-1 cells.

Ceramide is reported to activate 97 kDa serine/threonine kinase, termed ceramide-activated protein kinase (Kolesnick and Golde, 1994). In the present study, we found that treatment of parental cells with TNF-α or ceramide resulted in activation of PK35 (Figure 3). PK35 and ceramide-activated protein kinase can use MBP as an *in vitro* substrate. Liu *et al*. (1994) reported that treatment of HL-60 cells with TNF resulted in activation of ceramide-activated protein kinase between 5 and 10 min. In the present study, we noted that PK35 is activated around 60 min after TNF treatment. Hence, in size and kinetics of activation, PK35 may differ from ceramide-activated protein kinase.

In conclusion, TNF-α stimulates several serine/threonine kinases including PK50 and PK35 in parental Kasumi-1 cells. PK35 may prove to be a new member of the ceramide-activated protein kinase family and the lack of the activation of PK35 may be related to insensitivity of the variant cells to TNF-α.

**Acknowledgements**
We thank K Kita (2nd Department of Internal Medicine, Mie University) for providing us with the Kasumi-1 cell line and M Ohara for comments on the manuscript. This study was supported in part by a grant-in-aid from the Ministry of Education, Science and Culture of Japan.

### References

ASOU H, TASHIRO S, HAMAMOTO K, OTSUJI A, KITA K AND KAMADA N. (1991). Establishment of a human acute myeloid leukemia cell line (Kasumi-1) with 8;21 chromosome translocation. *Blood*, **77**, 2031–2036.

BEUTLER B AND CERAMI A. (1989). The biology of cachectin/TNF-A primary mediator of the host response. *Annu. Rev. Immunol*., **7**, 625–655.

COOPER JA, SEFTON BM AND HUNTER T. (1983). Detection and quantification of phosphotyrosine in proteins. *Methods Enzymol*., **99**, 387–402.

GOTOH Y, MORIYAMA K, MATSUDA S, OKUMURA E, KISHIMOTO T, KAWASAKI H, SUZUKI K, YAHARA I, SAKAI H AND NISHIDA E. (1991). *Xenopus* M phase MAP kinase: isolation of its cDNA and activation by MPF. *EMBO J*., **10**, 2661–2668.

ISHIYAMA M, SHIGA M, SASAMOTO K, MIZUGUCHI M AND HE P. (1993). A new sulfonated tetrazolium salt that produces a highly water-soluble formazan dye. *Chem. Pharm. Bull.*, **41**, 1118–1122.

ITOH N, YONEHARA S, ISHII A, YONEHARA M, MIZUSHIMA S, SAMESHIMA M, HASE A, SETO Y AND NAGATA S. (1991). The polypeptide encoded by the cDNA for human cell surface antigen Fas can mediate apoptosis. *Cell*, **66**, 233–243.

KAMESHITA I AND FUJISAWA H. (1989). A sensitive method for detection of calmodulin-dependent protein kinase II activity in sodium dodecyl sulfate-polyacrylamide gel. *Anal. Biochem.*, **183**, 139–143.

KIM M, LINARDIC C, OBEID L AND HANNUN Y. (1991). Identfication of sphingomyelin turnover as an effector mechanism for the action of tumor necrosis factor α and γ-interferon. *J. Biol. Chem.*, **266**, 484–489.

KOLESNICK R AND GOLDE DW. (1994). The sphingomyelin pathway in tumor necrosis factor and interleukin-1 signaling. *Cell*, **77**, 325–328.

KYRIAKIS JM, BANERJEE P, NIKOLAKAKI E, DAI T, RUBIE EA, AHMAD MF, AVRUCH J AND WOODGETT JR. (1994). The stress-activated protein kinase subfamily of c-Jun kinases. *Nature*, **369**, 156–160.

LINT JV, AGOSTINIS P, VANDEVOORDE V, HAEGEMAN G, FIERS W, MERLEVEDE W AND VANDENHEEDE JR. (1992). Tumor necrosis factor stimulates multiple serine/threonine protein kinases in Swiss 3T3 and L929 cells. *J. Biol. Chem.*, **267**, 25916–25921.

LIU J, MATHIAS S, YANG Z AND KOLESNICK RN. (1994). Renaturation and tumor necrosis factor-α stimulation of a 97 kDa ceramide-activated protein kinase. *J. Biol. Chem.*, **269**, 3047–3052.

LOETSCHER H, PAN YE, LAHM HW, GENTZ R, BROCKHAUS M, TABUCHI H AND LESSLAUER W. (1990). Molecular cloning and expression of the human 55 kd tumor necrosis factor receptor. *Cell*, **61**, 351–359.

OLD LJ. (1985). Tumor necrosis factor (TNF). *Science*, **230**, 630–632.

SCHALL TJ, LEWIS M, KOLLER KJ, LEE A, RICE GC, WONG GHW, GATANAGA T, GRANGER GA, LENTZ R, RAAB H, KOHR WJ AND GOEDDEL DV. (1990). Molecular cloning and expression of a receptor for human tumor necrosis factor. *Cell*, **61**, 361–370.

SMITH CA, DAVIS T, ANDERSON D, SOLAM L, BECKMANN MP, JERZY R, DOWER SK, COSMAN D AND GOODWIN RG. (1990). A receptor for tumor necrosis factor defines an unusual family of cellular and viral proteins. *Science*, **248**, 1019–1023.

TARTAGLIA LA, AYRES TM, WONG GHW AND GOEDDEL DV. (1993). A novel domain within the 55 kd TNF receptor signals cell death. *Cell*, **74**, 845–853.

VIETOR I, SCHWENGER P, LI W, SCHLESSINGER J AND VILCEK J. (1993). Tumor necrosis factor-induced activation and increased tyrosine phosphorylation of mitogen-activated protein (MAP) kinase in human fibroblasts. *J. Biol. Chem.*, **268**, 18994–18999.

VILCEK J AND LEE TH. (1991). Tumor necrosis factor. *J. Biol. Chem.*, **266**, 7313–7316.

WASHIO S, IDO M, AZUMA E, FUKUI M, SHIBATA T, ZANG S, KOMADA Y, ITO M, KOIKE T, KANEKO Y AND SAKURAI M. (1992). Acute megakaryoblastic leukemia with translocation t(1;22)(p13;q13) in a 10-week-old infant. *Am. J. Hematol.*, **39**, 56–60.